# Supplementary material for: TNFα-Induced LDL Cholesterol Accumulation Involve Elevated LDLR Cell Surface Levels and SR-B1 Downregulation in Human Arterial Endothelial Cells
Source: Int J Mol Sci. 2021 Jun 9;22(12):6236. doi: 10.3390/ijms22126236 (PMC8227244; doi:10.3390/ijms22126236)
Supplement: Supplementary file 1 [file ijms-22-06236-s001.zip › ijms-1244701-supplementary.pdf]

Table S1. List of Items Used

**Reagents**	**Company**	**Catalog number**
[^{3}H]Cholesterol	Perkin Elmer, Waltham, MA, U.S.A	NET139001
3 µm polyester membrane	Thermo Fisher Scientific, Waltham, MA, U.S.A.	3415
ABCA1 antibody	Santa Cruz Biotechnology, Dallas, Texas, U.S.A.	sc-58219
Antibiotic-antimycotic	Thermo Fisher Scientific, Waltham, MA, U.S.A.	15240112
Anti-goat-HRP	R&D Systems, Minneapolis, MN, U.S.A.	NL001
ApoE antibody	Santa Cruz Biotechnology, Dallas, Texas, U.S.A.	sc-13521
ApoE-conjugated agarose	Santa Cruz Biotechnology, Dallas, Texas, U.S.A.	sc-13521 AC
ASK 1 inhibitor	Tocris, Minneapolis, MN, U.S.A.	MSC 2032964A
BCA assay kit	Thermo Fisher Scientific, Waltham, MA, U.S.A.	23227
Bond elut column	Agilent Technologies, Santa Clara, CA, U.S.A.	12256060
Caspase inhibitor	Tocris, Minneapolis, MN, U.S.A.	2163
Caveolin-1 antibody	Santa Cruz Biotechnology, Dallas, Texas, U.S.A.	sc-53564
Centrifugal filter	Pall Corporation, New York, NY, U.S.A.	MAP003C36
Cholesterol esterase	MP Biomedicals Irvine, CA, U.S.A.	105439
Cholesterol oxidase	Sigma-Aldrich, St. Louis, MO, U.S.A.	C8649
Desalting columns	Thermo Fisher Scientific, Waltham, MA, U.S.A.	89890
Dialysis membrane	Spectrum Chemical, New Brunswick, NJ, U.S.A.	131204
Dil	Sigma-Aldrich, St. Louis, MO, U.S.A.	468495
EZ-link-Biotin	Thermo Fisher Scientific, Waltham, MA, U.S.A.	A39258
FAF-BSA	Sigma-Aldrich, St. Louis, MO, U.S.A.	A8806
FBS	R&D Systems, Minneapolis, MN, U.S.A.	S11550
Glass bottom dishes	Cellvis, Mountain View, CA, U.S.A.	P96-0-N
HRP	Alfa Aesar, Haverhill, MA, U.S.A.	J60026
ICAM1 antibody	Santa Cruz Biotechnology, Dallas, Texas, U.S.A.	sc-107
Iodination beads	Thermo Fisher Scientific, Waltham, MA, U.S.A.	28665
JNK inhibitor	Sigma-Aldrich, St. Louis, MO, U.S.A.	420118
LDLR antibody	R&D Systems, Minneapolis, MN, U.S.A.	AF2148
mHBSS	Sigma-Aldrich, St. Louis, MO, U.S.A.	H8264
NF-kB Inhibitor	Sigma-Aldrich, St. Louis, MO, U.S.A.	481412
NF-kB Inhibitor	Tocris, Minneapolis, MN, U.S.A.	4590
Normal goat IgG	R&D Systems, Minneapolis, MN, U.S.A.	AB-108-C
Normal mouse Ig agarose	Santa Cruz Biotechnology, Dallas, Texas, U.S.A.	sc-2343
p38 inhibitor	Sigma-Aldrich, St. Louis, MO, U.S.A.	506163
PCSK9	Caymen Chemical Ann Arbor, MI, U.S.A.	20631
pHAECs	ATCC, Manassas, VA, U.S.A.	PCS-100-011
Phosphatase inhibitor	Sigma-Aldrich, St. Louis, MO, U.S.A.	P0044
Protease inhibitor	Sigma-Aldrich, St. Louis, MO, U.S.A.	P8340
RAP	Enzo Life Sciences, Farmingdale, NY, U.S.A.	BML-SE552-0100
Sandoz 58-035	Sigma-Aldrich, St. Louis, MO, U.S.A.	S9318
Scopoletin	TCI America Portland, OR, U.S.A.	S0367

Sodium heparin	Thermo Fisher Scientific, Waltham, MA, U.S.A.	J16920-EXT
Sodium taurocholate	Beantown Chemicals, Hudson, NH, U.S.A.	141920
SR-B1	Novus Biologicals, Littleton, CO, U.S.A.	NB400-104
Strepavidin	GE Healthcare, Chicago, IL, U.S.A.	28-9872-30
TMTU	Alfa Aesar, Haverhill, MA, U.S.A.	L13392
TNFα	ProSci Inc, Poway, CA, U.S.A.	96-734
Trypsin-EDTA	Thermo Fisher Scientific, Waltham, MA, U.S.A.	25200-056
VBM	ATCC, Manassas, VA, U.S.A.	PCS-100-030
VEGF	ATCC, Manassas, VA, U.S.A.	PCS-100-041
VWF	Abcam, Cambridge, MA, U.S.A.	ab174290

Enhanced Uptake of LDL Mediated by TNFa Does Not Require Fully Confluent pHAECs

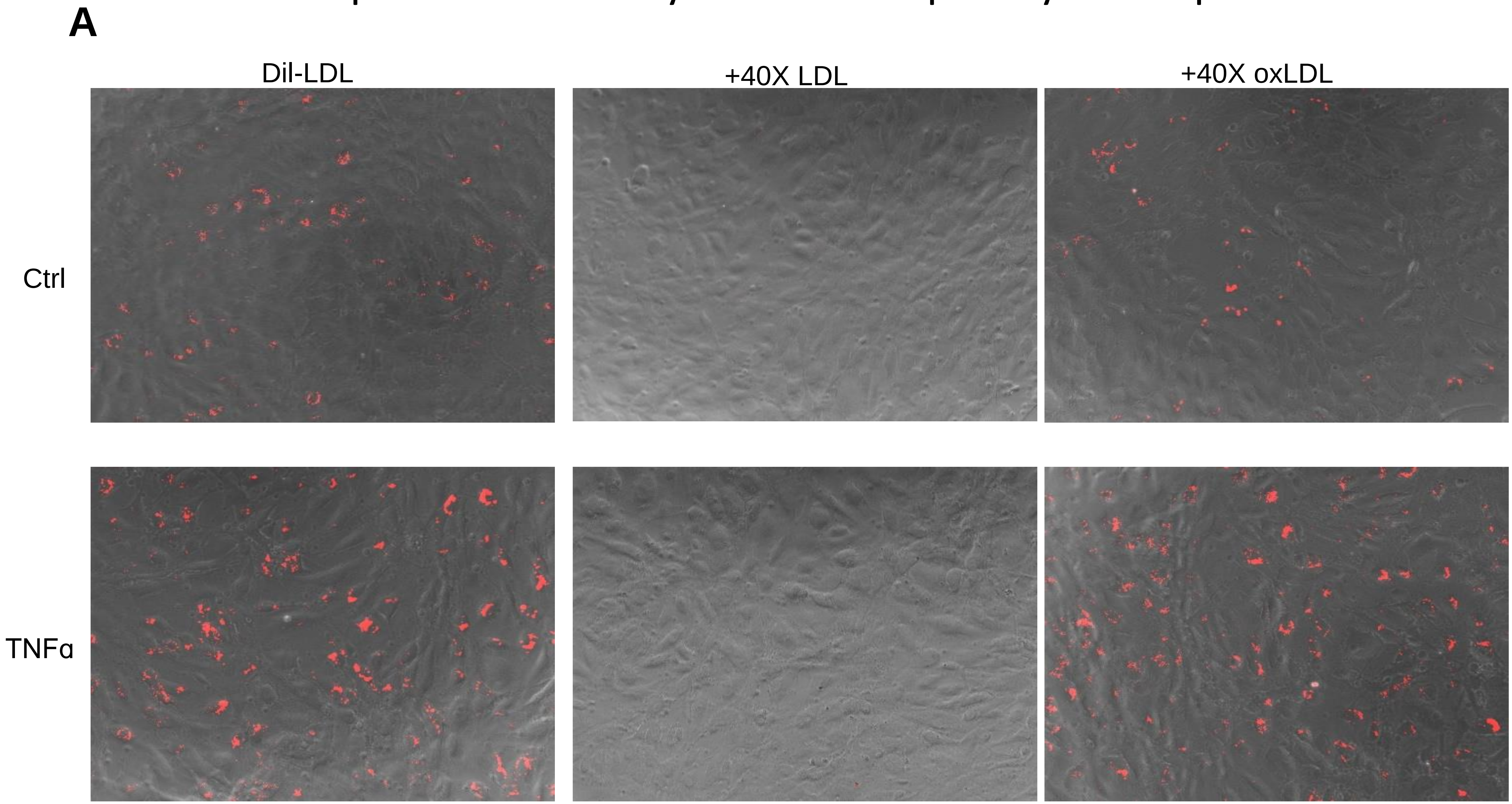

Figure S1

Original blots to Figure 1

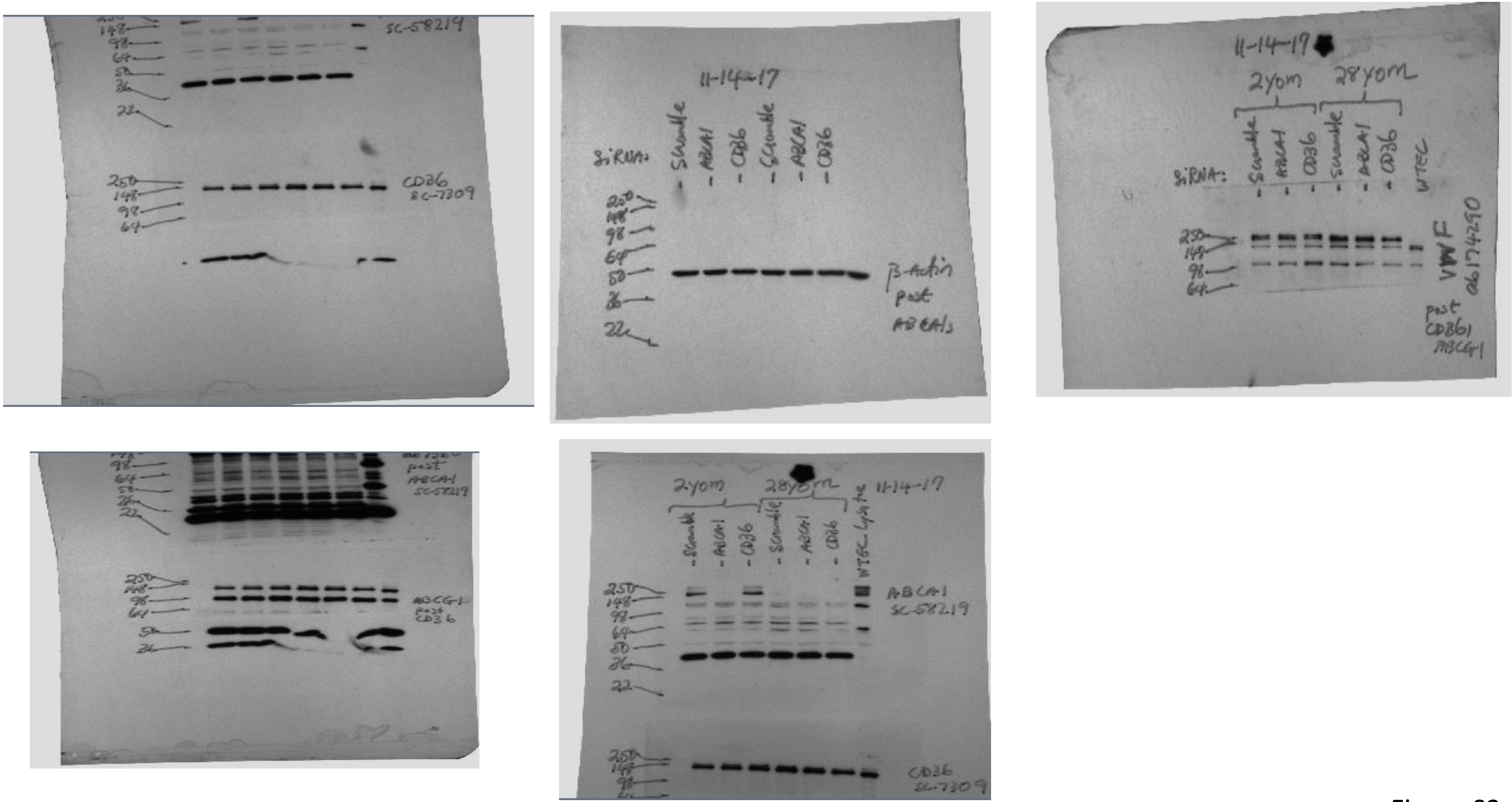

Figure S2

Original blots to Figure 6

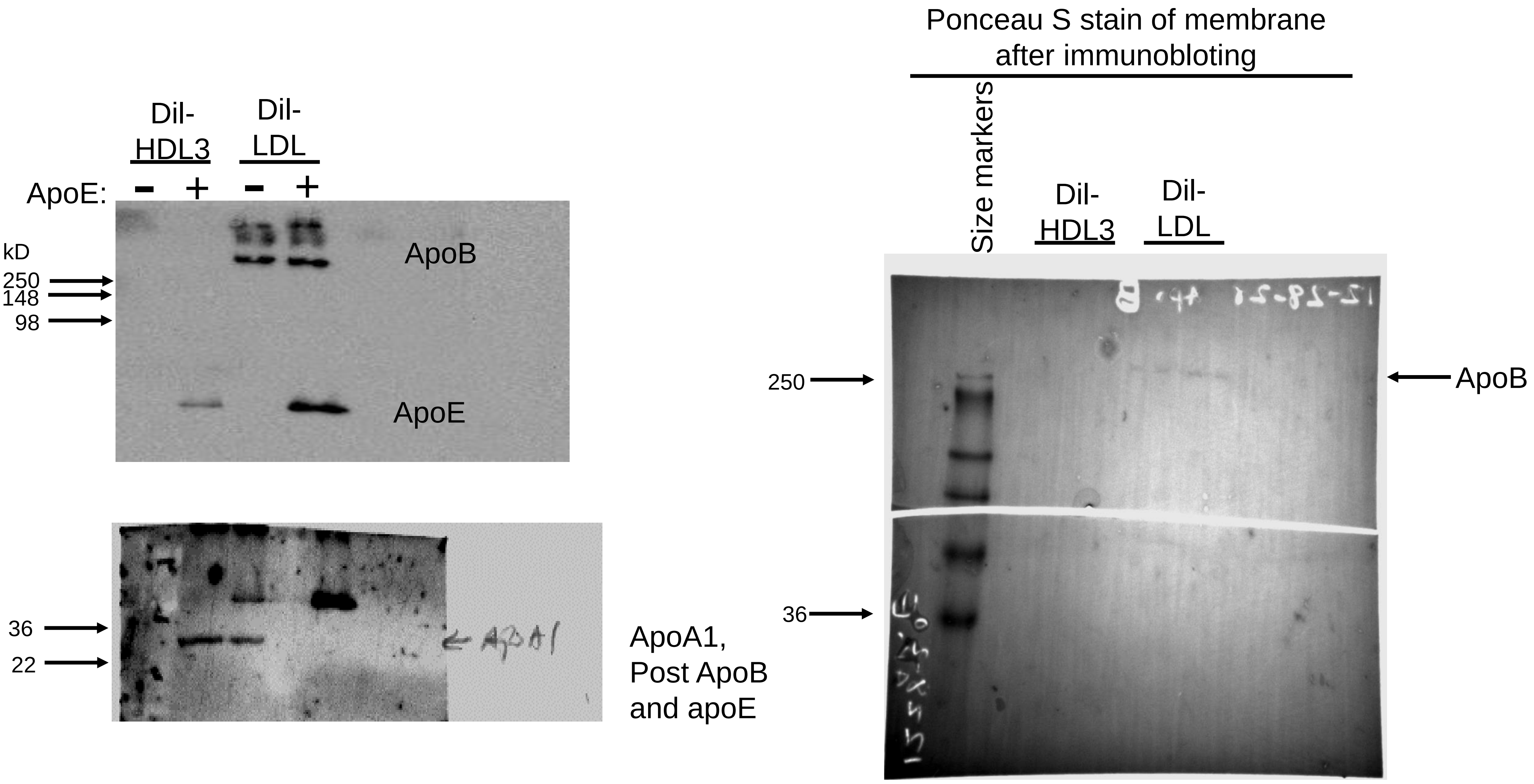

Figure S3

Supporting Information towards Figure 7

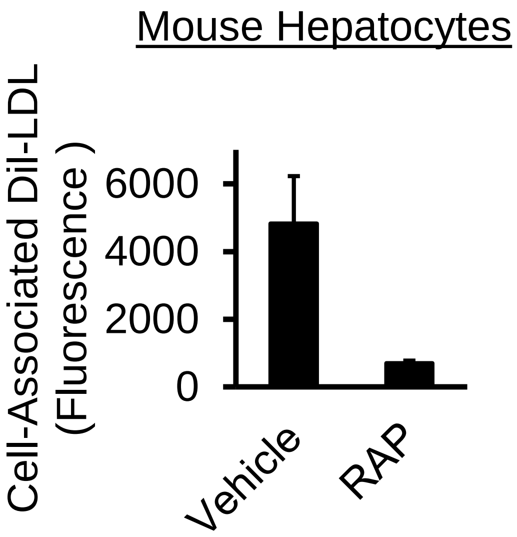

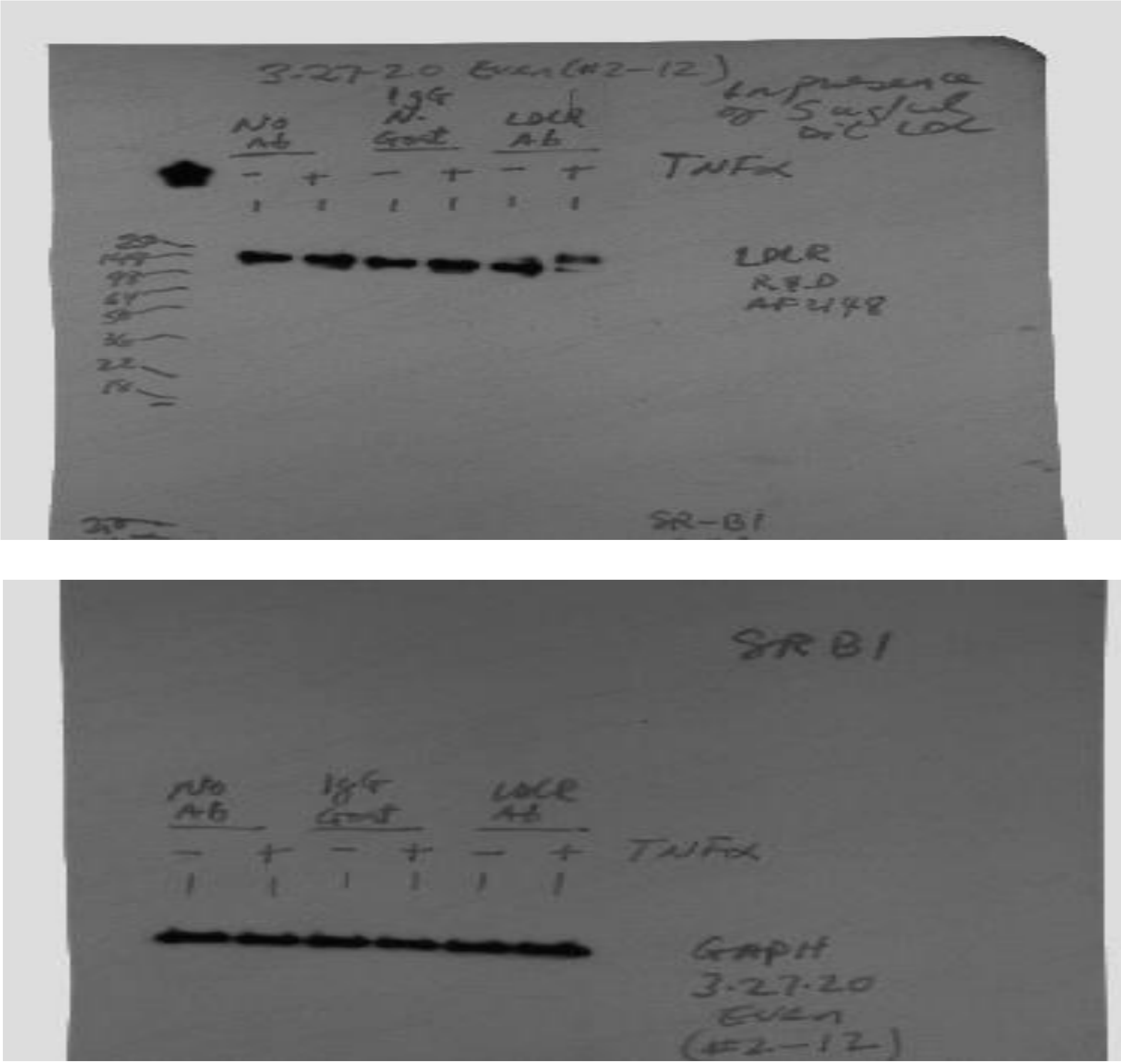

LDLR

GAPDH

Figure S4

Original blots to Figure 8 from the same lysates

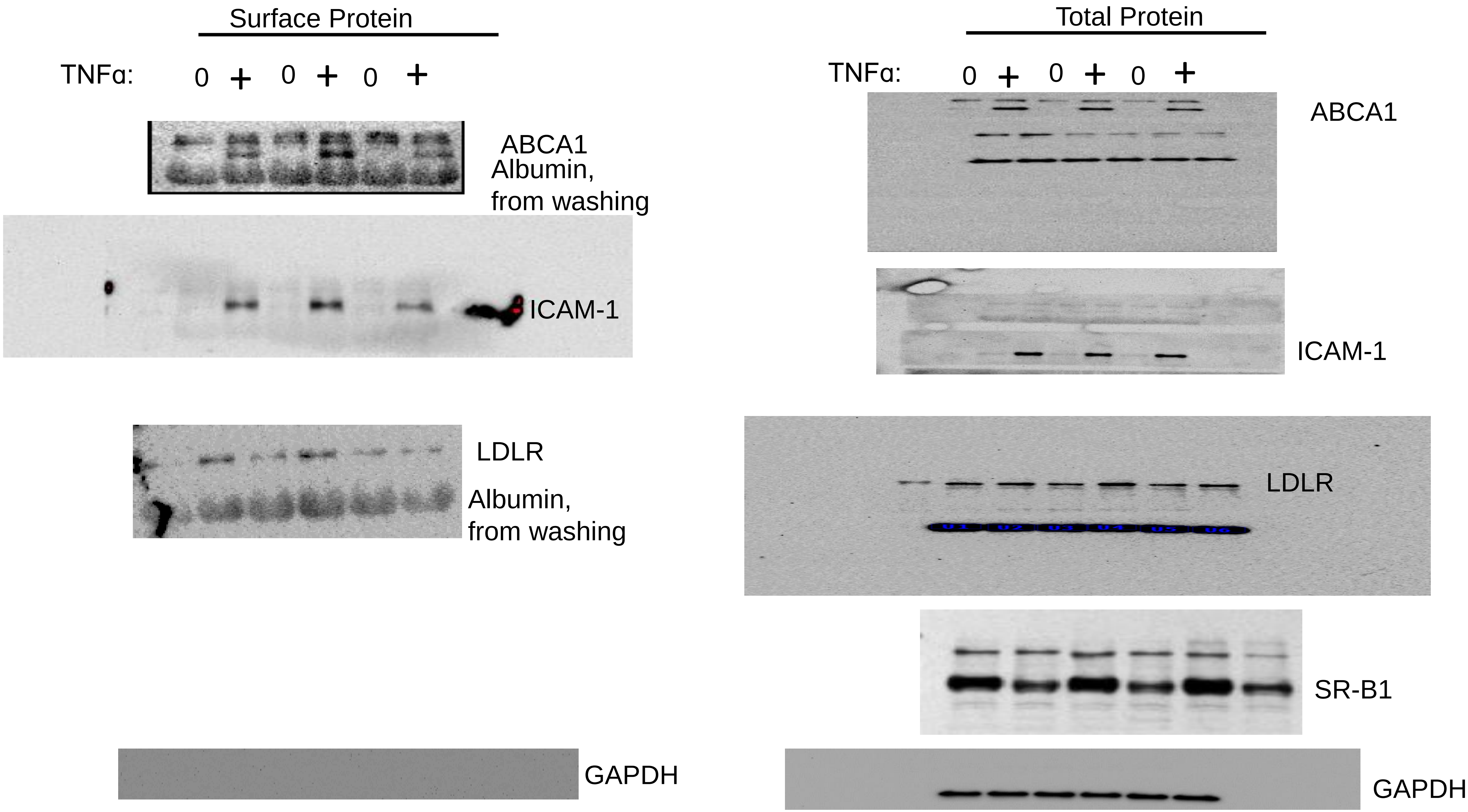

Figure S5
